# BEREAVED DEMENTIA CAREGIVERS MAKE MEANING THROUGH RELIGION IN A PHOTO-ELICITATION INTERVENTION

**DOI:** 10.1093/geroni/igae098.3810

**Published:** 2024-12-31

**Authors:** Annabelle Yang, Taryn Bogdewiecz, Jacquelyn Benson, Karla Washington, Abigail Rolbiecki

**Affiliations:** Washington University in St. Louis, St. Louis, Missouri, United States; University of Colorado Anschutz Medical Campus, Aurora, Colorado, United States; Washington University in St. Louis, St. Louis, Missouri, United States; Washington University in St. Louis, St. Louis, Missouri, United States; University of Colorado Anschutz, Aurora, Colorado, United States

## Abstract

Many persons living with dementia (PLWD) rely on informal caregivers, who undergo extensive personal upheaval approaching and after end-of-life for their PLWD. To promote successful adaptation in bereavement, caregivers can engage in a process of meaning making as they make sense of their loss, find “silver linings” in their experiences, or undergo shifts in identity. Religious and spiritual coping can facilitate this meaning making. Prior evidence has found that successful meaning making improves bereavement outcomes, reducing risk for grief complications. Caregiver Speaks is an intervention designed to facilitate such meaning making in caregivers of PLWD through photo-elicitation, whereby hospice family caregivers share stories and images narrating their experiences within a private Facebook group during active caregiving and bereavement. The purpose of the current study was to explore how caregivers from the bereavement arm of the Caregiver Speaks project made meaning of their experiences. Participants were recruited from hospices across the Midwest and Northeast US. A deductive and inductive qualitative analysis of Facebook posts was performed by two independent coders. They engaged in consensus coding to refine and categorize codes into themes. Religious and spiritual coping was a primary theme, as caregivers expressed feelings of peace knowing their loved ones were in Heaven, hope for the future because God has a plan, and comfort in scripture. Others described inner peace found in nature or meditative practices that provided comfort. These findings hold promise for meaning making interventions designed to facilitate religious and spiritual coping to improve bereavement outcomes.

